# Reference Intervals for 24-Hour Urinary Calcium Excretion and Its Association With Bone Metabolism: A Multicenter Study

**DOI:** 10.1210/clinem/dgae805

**Published:** 2025-02-28

**Authors:** Li Shen, Hao Zhang, Qi Lu, Shanshan Li, Yazhao Mei, Chao Gao, Hua Yue, Xiangtian Yu, Qi Yao, Yanan Huo, Yuhong Zeng, Yin Jiang, Zhongjian Xie, Aijun Chao, Xiaolan Jin, Guangjun Yu, Li Mao, Zhenlin Zhang

**Affiliations:** School of Public Health, Shanghai Jiao Tong University School of Medicine, Shanghai 200225, China; Department of Osteoporosis and Bone Disease, Shanghai Clinical Research Center of Bone Disease, Sixth People’s Hospital Affiliated to Shanghai Jiao Tong University School of Medicine, Shanghai 200233, China; Clinical Research Center, Sixth People’s Hospital Affiliated to Shanghai Jiao Tong University School of Medicine, Shanghai 200233, China; Department of Osteoporosis and Bone Disease, Shanghai Clinical Research Center of Bone Disease, Sixth People’s Hospital Affiliated to Shanghai Jiao Tong University School of Medicine, Shanghai 200233, China; Department of Osteoporosis and Bone Disease, Shanghai Clinical Research Center of Bone Disease, Sixth People’s Hospital Affiliated to Shanghai Jiao Tong University School of Medicine, Shanghai 200233, China; Department of Osteoporosis and Bone Disease, Shanghai Clinical Research Center of Bone Disease, Sixth People’s Hospital Affiliated to Shanghai Jiao Tong University School of Medicine, Shanghai 200233, China; Department of Osteoporosis and Bone Disease, Shanghai Clinical Research Center of Bone Disease, Sixth People’s Hospital Affiliated to Shanghai Jiao Tong University School of Medicine, Shanghai 200233, China; Department of Osteoporosis and Bone Disease, Shanghai Clinical Research Center of Bone Disease, Sixth People’s Hospital Affiliated to Shanghai Jiao Tong University School of Medicine, Shanghai 200233, China; Department of Osteoporosis and Bone Disease, Shanghai Clinical Research Center of Bone Disease, Sixth People’s Hospital Affiliated to Shanghai Jiao Tong University School of Medicine, Shanghai 200233, China; Clinical Research Center, Sixth People’s Hospital Affiliated to Shanghai Jiao Tong University School of Medicine, Shanghai 200233, China; Department of Geriatrics, The First Affiliated Hospital of Ningbo University, Ningbo 315010, China; Department of Endocrinology, Jiangxi People’s Hospital, Nanchang 331400, China; Department of Osteoporosis, Honghui Hospital Affiliated to Xi’an Jiaotong University, Xi’an 710054, China; Department of Rheumatism, Liuzhou Worker’s Hospital, Liuzhou 545000, China; Hunan Provincial Key Laboratory of Metabolic Bone Diseases, National Clinical Research Center for Metabolic Diseases, Department of Metabolism and Endocrinology, The Second Xiangya Hospital of Central South University, Changsha 410011, China; Department of Orthopaedics, Tianjin Hospital, Tianjin 300211, China; Department of Endocrinology, Chengdu Military General Hospital, Chengdu 610075, China; School Engineering Research Center for Big Data in Pediatric Precision Medicine, Shanghai 200040, China; School of Medicine, The Chinese University of HongKong, Shenzhen 518000, China; Department of Endocrinology, Huai’an First People’s Hospital Affiliated to Nanjing Medical University, Huai’an 223300, China; Department of Osteoporosis and Bone Disease, Shanghai Clinical Research Center of Bone Disease, Sixth People’s Hospital Affiliated to Shanghai Jiao Tong University School of Medicine, Shanghai 200233, China; Clinical Research Center, Sixth People’s Hospital Affiliated to Shanghai Jiao Tong University School of Medicine, Shanghai 200233, China

**Keywords:** 24-hour urinary calcium excretion, reference intervals, bone metabolism, multicenter cross-sectional study

## Abstract

**Context:**

The 24-hour urinary calcium excretion (UCaE) not only serves as an important indicator of calcium metabolism balance but also correlates with metabolic diseases. However, the distribution of 24-hour UCaE and its relationship with bone metabolism are unknown.

**Objective:**

To investigate the distribution of 24-hour UCaE and its association with bone metabolism.

**Methods:**

In this multicenter cross-sectional study, 1239 participants underwent physical examinations at 9 tertiary hospitals. Multivariate linear regression was used to explore bone metabolism associated with 24-hour UCaE. The relationship of bone metabolism with 24-hour urinary calcium excretion/urinary creatinine (UCaE/Ucr) was analyzed by using restrictive cubic splines fitting multiple linear regression model.

**Results:**

The 24-hour UCaE median range was 2.27 mmol overall, 2.24 mmol in men, and 2.28 mmol in women. For men, the highest 24-hour UCaE/Ucr was observed in those aged between 30 and 44 years (median: 0.70), whereas the lowest was found aged between 18 and 29 years (median: 0.46). For women, the 24-hour UCaE/Ucr showed a gradual increase with advancing age. In the adjusted model, 24-hour UCaE/Ucr was independently positively associated with 25(OH)D in both men (*P* = .032) and women (*P* < .001). It was independently associated with parathyroid hormone (PTH) (*P* = .031), type Ⅰ collagen-containing cross-linked C-telopeptide (β-CTX) (*P* = .021) and procollagen type I N-propeptide (P1NP) (*P* = .048) in men, but not in women. The prevalence of hypercalciuria was 11.6% (men 7.5%; women 14.0%) and significantly varied across age groups and regions (*P* < .05).

**Conclusion:**

This study established reference intervals for 24-hour UCaE in the Chinese population. This study found gender differences in the relationship between 24-hour UCaE and bone metabolism.

Calcium plays a key role in a variety of physiological processes (eg, cell excitability, muscle contraction, and bone formation) (1). Tissue calcium levels are tightly regulated through a dynamic process (2). Insufficient calcium intake and excessive clearance over a prolonged period lead to calcium loss in the body, increasing the risk of osteoporosis and fractures (3). Calcium is primarily cleared through renal excretion, with minor contributions from fecal and dermal losses. As the most abundant mineral in the body, calcium is crucial for bone health and essential for cardiovascular, muscular, and nervous system function (2). Urinary calcium is derived from the filtration of albumin-free plasma calcium and subsequent reabsorption across the renal tubules (4). The 24-hour urinary calcium excretion (UCaE) not only serves as an important indicator of calcium metabolism balance but also correlates with metabolic diseases. Elevated 24-hour UCaE indicates excessive calcium intake, poor reabsorption, or increased bone resorption, and is associated with conditions like osteoporosis or hyperparathyroidism (4). Conversely, low 24-hour UCaE suggests insufficient intake and is related to vitamin D deficiency or chronic kidney disease (5, 6). The assessment of 24-hour UCaE is integral in managing osteoporosis, lithogenesis and bone metabolism disorders (7). The determination of urinary calcium is a common clinical test used for patients with various calcium and bone metabolism disorders. The UCaE is influenced by calcium intake and absorption, which vary among different ethnic groups. Differences in food availability, cultural preferences and socioeconomic factors contribute to differences in dietary intake and calcium absorption between ethnic groups within and between countries (8, 9). Since calcium excretion is determined by intake and absorption, studying excretion levels becomes particularly important. Currently, there is a lack of large-scale, multicenter studies on 24-hour UCaE in Chinese population. Establishing reference intervals specific to the Chinese adult population for 24-hour UCaE distribution is currently lacking, which is crucial for identifying metabolic abnormalities, guiding clinical decisions, and informing public health strategies (10).

Bone is a mineralized connective tissue in which calcium is the primary component, providing bone strength and structure (11). Previous studies have shown a negative correlation between 24-hour UCaE and cortical bone mineral density (BMD) (12). In clinical trials and routine clinical practice, serum concentrations of bone turnover markers (BTMs) are widely used to assess the treatment response to anti-osteoporosis medications (13). Compared to BMD, measurement of BTMs can reflect changes in bone remodeling within a short timeframe and contribute independently to fracture risk prediction (14, 15). Understanding the relationship between 24-hour UCaE and BTMs not only provides insight into the relationship between calcium levels and bone health, but also facilitates assessment of individual calcium metabolism (16). Investigating the relationship between 24-hour UCaE and BTMs can enhance our understanding of bone metabolism mechanisms, providing a theoretical basis for diagnosing and treating skeletal and metabolic diseases. However, research on this relationship is currently limited. With the rapid social and economic development, lifestyle changes have led to increased calcium and vitamin D supplementation compared to earlier periods in China (17). Epidemiological data on 24-hour UCaE and its association between 24-hour UCaE and BTMs are scant.

To date, there have been no large-scale, representative studies of 24-hour UCaE in China. Establishing reference intervals for 24-hour UCaE in the Chinese adult population is crucial for identifying metabolic abnormalities, guiding clinical decisions, and informing public health strategies. We conducted a multicenter cross-sectional study to investigate the distribution of 24-hour UCaE by sex and age in China, and to investigate the relationship between 24-hour UCaE and parathyroid hormone (PTH), 25-hydroxyvitamin D [25(OH)D], and BTM levels.

## Methods

### Study Participants and Setting

This multicenter, cross-sectional study (CHinA National Calcium lEvel Survey [CHANCES]), conducted between March 2022 and March 2023, enrolled adult participants (aged > 18 years) at 9 tertiary care hospitals in China. We excluded participants with uremia, type 1 diabetes mellitus, Cushing syndrome, hyperthyroidism, hyperparathyroidism, hypothyroidism, active cancer, and chronic kidney disease. Participants who had autoimmune disease within the preceding 3 months and required glucocorticoids were ineligible. Other exclusion criteria were recent use of diuretics or other drugs that could affect urinary calcium levels. Pregnant or lactating women were excluded.

The study protocol was approved by the Ethics Committee of Shanghai Sixth People's Hospital. This study was registered with CHICTR.ORG.CN (ChiCTR2200056577) and performed in accordance with the Declaration of Helsinki. All participants provided written informed consent before the study.

### Data Collection

A customized questionnaire was used to collect demographic information, current alcohol consumption, and current smoking status. Alcohol consumption was defined as present if participants reported consuming alcohol. Smoking status was divided into current smoker and nonsmoker based on participant reports. Additionally, the questionnaire gathered history of diabetes mellitus, hypertension, or use of antiglycemic drugs, antihypertensive drugs, and calcium and vitamin D supplements. Hypertension was defined as systolic blood pressure > 140 mmHg, diastolic blood pressure of > 90 mmHg, or current use of antihypertensive agents. All participants were measured for height and weight using standardized methods, with light clothing and no shoes during the measurements.

### Urine Collections

Participants received comprehensive instructions for urine specimen collection. Professional researchers instructed the participants and their relatives on the method of 24-hour urine collection and distributed printed instruction manuals and records. Each participant received 3 to 5 grams of benzoic acid (preservative), a 5-L urine container, a stirring rod, a dropper, and three 10-mL urine tubes (18). The participants were instructed to collect in the container all the urine produced in 24 hours, and to make the maximum effort to avoid dispersing urine during the collection period. Time of start and end of urine collection and episodes of urine dispersion had to be annotated in the records. The urine was then to be thoroughly mixed using the stirring rod, transferred into tubes, and brought to the clinic at the next visit. The urine sample tubes were labeled, stored at −80 °C and sent to the central laboratory for testing within the valid timeframe. Seasonality of urine sample collection was defined as spring (March to May), summer (June to August), autumn (September to November), and winter (December to February). When participants had urinary tract infections, menstruation, or fever, they were instructed to postpone urine collection.

### Laboratory Assays

Fasting blood specimens were collected during the second visit. All blood and urine specimens were tested at the central laboratory. Liver and kidney function tests, including alkaline phosphatase (ALP), alanine aminotransferase (ALT), uric acid, blood urea nitrogen (BUN), serum calcium, phosphorus, and urinary calcium (UCaE) were measured using the Cobas c701 automatic biochemical analyzers (Roche Diagnostics, Basel, Switzerland). BTMs were measured using electrochemiluminescence immunoassays using commercially available kits for procollagen type 1 N-propeptide (P1NP), type I collagen-containing cross-linked C-telopeptide (β-CTX), PTH, and vitamin D total kit for 25(OH)D as instructed by the manufacturer (Roche Diagnostics, Basel, Switzerland). Urinary creatinine (Ucr) was measured by the creatinine oxidase method using a creatinine kit (Roche Diagnostics, Basel, Switzerland) (19). Intra- and interpatch coefficients of variation for urinary values were 0.80% and 1.40% for concentrations of 14.60 and 18.41 mg/dL, respectively; 1.20% and 1.60% for concentrations of 6.23 and 6.15 mg/dL, respectively; 0.90% and 1.40% for concentrations of 11.03 and 10.91 mg/dL, respectively (4). Hypercalciuria was defined as 24-hour UCaE > 7.5 mmol in men and > 6.25 mmol in women (12, 20).

### Dietary Intake

Dietary calcium intake was assessed using a 1-week food frequency questionnaire (21). Participants were required to complete a comprehensive dietary questionnaire, detailing their food intake, portion sizes, and frequency of consumption on both a daily and weekly basis. To minimize significant fluctuations in 24-hour UCaE due to dietary changes, participants were instructed to maintain detailed dietary logs on the day of baseline urine collection and to adhere to their baseline diet as closely as possible during subsequent urine collection periods. The daily calcium intake per person was calculated with reference to the Chinese Food Composition Table (21).

### Statistical Analysis

Normality was tested using Shapiro-Wilk test. Normally distributed variables were expressed as the mean ± SD and independent *t* test was used for between-group comparisons, while non-normally distributed variables were expressed as the median (Q1, Q3) and Mann-Whitney U test was used for between-group comparisons. Categorical variables were expressed as frequency and percentages (%), and chi-square test was used for between-group comparisons. Because 24-hour UCaE and 24-hour UCaE/Ucr did not fit a normal distribution, the 5th, 10th, 25th, 50th, 75th 90th, and 95th percentiles of 24-hour UCaE and 24-hour UCaE/Ucr were calculated to display population distribution. To depict the distribution of 24-hour UCaE and 24-hour UCaE/Ucr, Kernel density plot was employed. This study used box plots to describe the distribution of 24-hour UCaE/Ucr by age, geographic region, and season, and the Wilcoxon-Mann-Whitney test was used to compare the difference of 24-hour UCaE/Ucr between different age, region, and season group. The 24-hour UCaE/Ucr was non-normally distributed and ln-transformed was used. Multivariable linear regression was used to explore the association between covariates of interest and 24-hour UCaE/Ucr. Covariates included age, body mass index (BMI), smoking, alcohol consumption, diet calcium, diabetes mellitus, hypertension, season, and medications known to affect calcium excretion (eg, calcium supplements and vitamin D supplements). The analysis was conducted using an unadjusted model and a model that adjusted with a *P* value of .10 as a covariate, along with age, BMI, calcium supplements, and vitamin D supplements. PTH, 25(OH)D, β-CTX, and P1NP were divided into quartiles. Subgroup analyses were stratified by age, menopausal status, BMI, dietary calcium intake, and calcium supplement. The dose-response relationship of PTH, 25(OH)D, β-CTX, and P1NP with 24-hour UCaE/Ucr were analyzed by using restrictive cubic splines fitting multiple linear regression model. This study used bar charts to describe the distribution of hypercalciuria by age, region, and season.

Unless stated otherwise, two-tailed *P* values of <.05 were considered statistically significant. All statistical analyses were performed using R software 4.3.0 (R Foundation for Statistical Computing, Vienna, Austria) and IBM SPSS (version 26.0; SPSS Inc, Chicago, IL, USA).

## Results

### Characteristics of the Study Population

Of 1289 participants screened for eligibility, 1239 participants including 786 (63.4%) women, were enrolled and included in the analysis. The baseline characteristics are shown in Table 1. In the overall population, mean age (± SD) was 48.0 ± 18.1 years, and the mean BMI (± SD) was 23.16 ± 4.20 kg/m^2^. There were 9.8% current smokers, and 9.4% with current alcohol consumption. There were 9.0% diabetes and 22.0% hypertensions among the participants and the median diet calcium was 255.81 mg. The median 24-hour UCaE in the study population was 2.27 mmol. Specifically, the median 24-hour UCaE was 2.24 mmol in men and 2.28 mmol in women. There was no significant difference between men and women in 24-hour UCaE (*P* = .355). Notably, the 24-hour UCaE was significantly lower in premenopausal women than postmenopausal women (2.01 mmol vs 2.70 mmol, *P* < .001). The 24-hour UCaE/Ucr was significantly lower in men than women (0.55 vs 0.79, *P* < .001). Men consumed more dietary calcium but less vitamin D supplementation than women (both *P* < .05). There were statistically significant differences between men and women in β-CTX, 25(OH)D, ALP, BUN, phosphorus, ALT, and uric acid (all *P* < .05) (Table 1). The distributions of 24-hour UCaE and 24-hour UCaE/Ucr were rightly skewed in both men and women (Supplementary Fig. S1 (22)).

**Table 1.** Participant characteristics by sex

Characteristics	Total (n = 1239)	Men (n = 453)	Women (n = 786)	*P* value
Age, years	48.00 ± 18.10	47.91 ± 17.99	48.06 ± 18.17	.891
Height, cm	163.27 ± 17.54	167.56 ± 22.13	160.80 ± 13.66	**<**.**001**
Weight, kg	62.46 ± 14.29	69.28 ± 15.30	58.54 ± 12.03	**<**.**001**
BMI, kg/m^2^	23.16 ± 4.20	24.28 ± 4.20	22.53 ± 4.07	**<**.**001**
Current smokers	122 (9.8)	110 (24.3)	12 (1.5)	**<**.**001**
Alcohol consumption	116 (9.4)	94 (20.8)	22 (2.8)	**<**.**001**
Diabetes mellitus, n (%)	112 (9.0)	40 (8.8)	72 (9.2)	.845
Hypertension, n (%)	272 (22.0)	89 (19.6)	183 (23.3)	.137
Diet calcium, mg	255.81 (116.25, 473.10)	273.30 (145.71, 505.61)	243.35 (101.31, 435.53)	.**001**
Calcium supplementation	214 (17.3)	72 (15.9)	142 (18.1)	.330
Vitamin D supplementation	213 (17.2)	55 (12.1)	158 (20.1)	**<**.**001**
Season				.**004**
Spring + Winter	339 (27.4)	146 (32.2)	193 (24.6)	
Summer + Autumn	900 (72.6)	307 (67.8)	593 (75.4)	
Hypercalciuria (n,%)	144 (11.6)	34 (7.5)	110 (14.0)	**<**.**001**
24-hour UCaE, mmol	2.27 (1.31, 4.06)	2.24 (1.23, 4.17)	2.28 (1.33, 3.98)	.355
24-hour UCaE/Ucr	0.69 (0.36, 1.37)	0.55 (0.29, 0.99)	0.79 (0.42, 1.76)	**<**.**001**
PTH, ng/L	35.60 (27.75, 45.45)	35.70 (27.60, 46.20)	35.55 (27.80, 45.16)	.969
β-CTX, ng/mL	0.38 (0.25, 0.56)	0.42 (0.29, 0.60)	0.36 (0.24, 0.53)	**<**.**001**
P1NP, ng/mL	42.90 (33.65, 58.15)	43.70 (32.40, 59.00)	42.60 (32.84, 58.13)	.736
25(OH)D, ng/mL	17.00 (12.00, 23.00)	18.00 (13.00, 24.00)	15.00 (11.00, 22.00)	**<**.**001**
Serum calcium, mmol/L	2.34 (2.25, 2.46)	2.33 (2.23, 2.45)	2.35 (2.26, 2.46)	.083
ALP, U/L	70.0 (55.0, 86.0)	76.0 (62.0, 89.0)	66.0 (53.0, 82.0)	**<**.**001**
BUN, mmol/L	4.50 (3.70, 5.50)	4.90 (4.10, 5.80)	4.30 (3.50, 5.10)	**<**.**001**
Phosphorus, mmol/L	1.21 (1.08, 1.36)	1.15 (1.02, 1.34)	1.25 (1.12, 1.38)	**<**.**001**
ALT, U/L	13 (9.00, 20.00)	17.00 (11.00, 25.00)	12.00 (8.00, 18.00)	**<**.**001**
UA, μmol/L	292.00 (242.00, 358.00)	353.00 (288.00, 414.00)	272.00 (230.75, 320.00)	**<**.**001**

Normally distributed variables were expressed as the mean ± SD and independent *t* test was used for between-group comparisons, while non-normally distributed variables were expressed as the median (Q1, Q3) and Mann-Whitney U test was used for between-group comparisons. Categorical variables were expressed as frequency and percentages (%), and chi-square test was used for between-group comparisons, unless otherwise specified.

*P* values for differences in baseline characteristics between sex groups. Bold entries are a significant value of *P* < .05.

Abbreviations: 25(OH)D, 25-hydroxyvitamin D; ALP, alkaline phosphatase; ALT, alanine aminotransferase; β-CTX, type Ⅰ collagen-containing cross-linked C-telopeptide; BMI, body mass index; BUN, blood urea nitrogen; P1NP, procollagen type I N-propeptide; PTH, parathyroid hormone; UA, uric acid; UCaE, urinary calcium excretion; Ucr, creatinine.

### Profile of 24-Hour UCaE/Ucr by Age, Region, and Season

**Figure 1.**
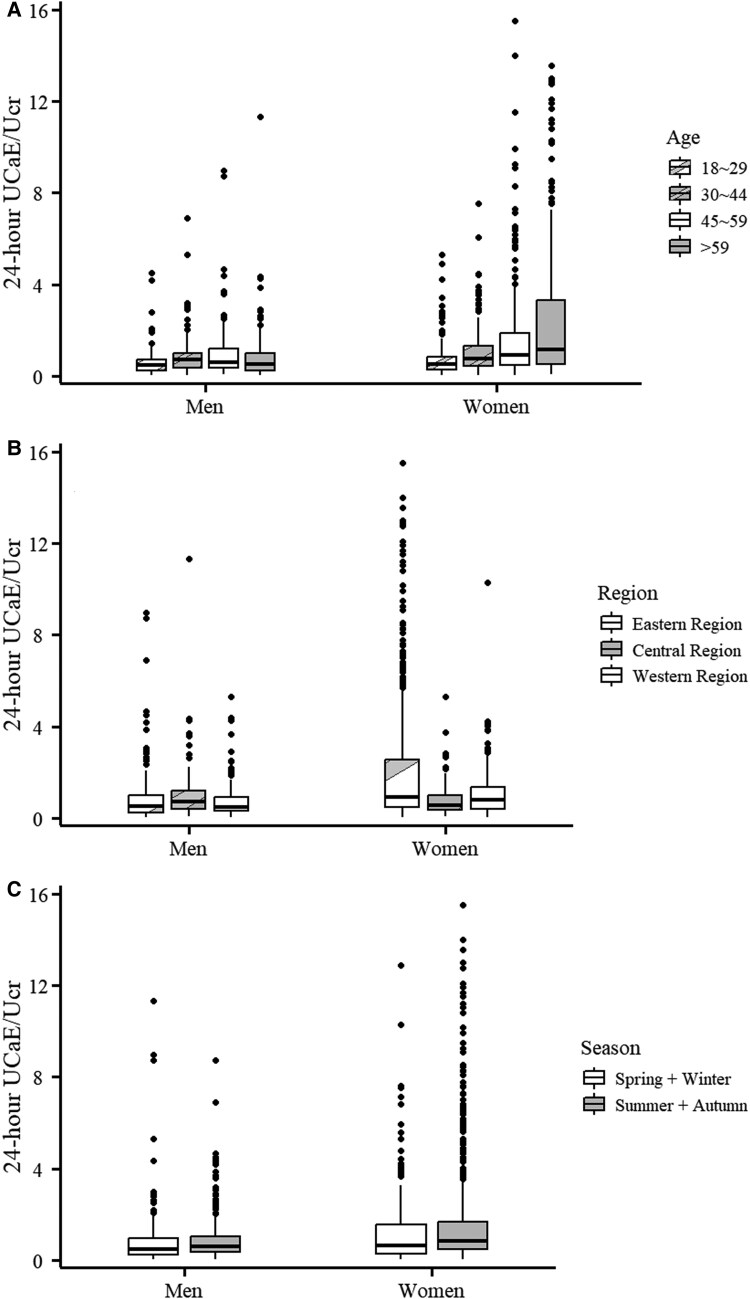
Distribution of 24-hour urinary calcium excretion/urinary creatinine (UCaE/Ucr) by age, region, and season. Box plot depicting the distribution of 24-hour UCaE/Ucr by sex. (A) Age; (B) Region; (C) Season.

There was a significant difference in the relationship between 24-hour UCaE/Ucr and age in both men (*P* = .011) and women (*P* < .001) (Fig. 1A). Reference values for 24-hour UCaE and UCaE/Ucr and of men and women at 15-year age intervals are provided in Supplementary Table S1a and Supplementary Table S1b (22). For 24-hour UCaE/Ucr, among men, the highest median was observed in those aged between 30 and 44 years (0.70, Q1-Q3: 0.36-1.01), whereas the lowest was found in men aged between 18 and 29 years (0.46, Q1-Q3: 0.24-0.74) (Fig. 1A and Supplementary Table S1 (22)). In contrast, among women, the 24-hour UCaE/Ucr showed a gradual increase with advancing age, from a median of 0.53 (Q1-Q3: 0.28-0.84) in those aged between 18 and 29 years 1.17 (Q1-Q3: 0.54-3.56) in those aged ≥ 60 years (Fig. 1A).

There was a statistically significant difference in the relationship between 24-hour UCaE/Ucr and region in both men (*P* = .017) and women (*P* < .001) (Fig. 1B). The highest 24-hour UCaE/Ucr among men is in the Central region (median 0.69, Q1-Q3: 0.39-1.23), followed by the Eastern region (median 0.53, Q1-Q3: 0.25-1.01), and the lowest is in the Western region (median 0.49, Q1-Q3: 0.29-0.94). However, for women, the highest 24-hour UCaE is in the Eastern region (median 0.92, Q1-Q3: 0.46-2.86), followed by the Western region (median 0.78, Q1-Q3: 0.38-1.38), and the lowest is in the Central region (median 0.56, Q1-Q3: 0.35-1.01). There was also statistically significant difference in the relationship between 24-hour UCaE/Ucr and season in both men (*P* = .028) and women (*P* = .001) (Fig. 1C). Both in men (0.47 vs 0.58) and women (0.65 vs 0.83), 24-hour UCaE/Ucr in spring and winter was significantly lower than in summer and autumn.

### Factors Associated With 24-Hour UCaE/Ucr

We performed multivariable regression analysis to investigate age, dietary calcium levels, seasons, vitamin D supplementation, serum calcium, and ALP as possible determinants factors of 24-hour UCaE/Ucr. The multivariable regression analysis is presented in **Table 2**. We observed that 24-hour UCaE/Ucr was independently associated with dietary calcium levels, hypertension, and season in both men and women after adjustment for covariates. Specifically, the 24-hour UCaE/Ucr was statistically positively associated with higher dietary calcium (both *P* < .05) in both men and women. Furthermore, the 24-hour UCaE/Ucr was significantly lower in the spring and winter compared to the summer and autumn in both men (β = .311, *P* = .003) and women (β = .364, *P* < .001). Vitamin D supplementation (β = .560, *P* < .001) was positively associated with 24-hour UCaE/Ucr, and ALP (β = −.004, *P* = .015) and phosphorus (β = −.355, *P* = .018) were negatively associated with 24-hour UCaE/Ucr in women.

**Table 2.** Factors associated with ln-transformed 24-hour UCaE/Ucr by sex

Independent variables	Men				Women			
	Model 1		Model 2		Model 1		Model 2	
	β (95% CI)	*P* value	β (95% CI)	*P* value	β (95% CI)	*P* value	β (95% CI)	*P* value
Age (per 10 y)	.024 (−.030, .077)	.386	.043 (−.017, .103)	.160	.201 (.159, .244)	**<**.**001**	.122 (.071, .173)	**<**.**001**
BMI (kg/m^2^)	−.006 (−.029, .016)	.579	−.003 (−.025, .020)	.805	.023 (.003, .043)	.**028**	−.014 (−.033, .006)	.168
Smoking (n,%)	.509 (.293, .725)	**<**.**001**	.395 (−.144, .647)	.**002**	−.065 (−.736, .606)	.849	.014 (−.601, .629)	.964
Drinking (n,%)	.399 (.168, .631)	.**001**	.209 (−.049, .466)	.112	−.511 (−.998, −.024)	.**040**	−.357 (−.813, .100)	.125
Dietary calcium (per 100 mg)	.029 (.010, .039)	.**003**	.031 (.011, .050)	.**002**	.064 (.042, .086)	**<**.**001**	.063 (.043, .084)	**<**.**001**
Diabetes (n,%)	.147 (−.188, .481)	.389	.060 (−.298, .418)	.742	.980 (.703, 1.256)	**<**.**001**	.215 (−.086, .515)	.161
Hypertension (n,%)	−.064 (−.302, .174)	.599	−.216 (−.492, .060)	.124	.932 (.749, 1.116)	**<**.**001**	.303 (.071, .535)	.**011**
Season	.226 (.023, .428)	.**029**	.311 (.110, .512)	.**003**	.355 (.165, .544)	**<**.**001**	.364 (.191, .536)	**<**.**001**
Calcium supplementation	−.222 (−.479, .034)	.089	−.312 (−.637, .013)	.060	.669 (.462, .876)	**<**.**001**	.033 (−.198, .263)	.791
Vitamin D supplementation	.111 (−.179, .402)	.452	−.245 (−.111, .602)	.177	.982 (.790, 1.175)	**<**.**001**	.560 (.337, .783)	**<**.**001**
Serum calcium (mmol/L)	.103 (−.211, .416)	.520	.133 (−.191, .457)	.420	−.081 (−.300, .137)	.466	.046 (−.154, .247)	.650
ALP (U/L)	−.001 (−.004, .003)	.778	.001 (−.003, .004)	.748	.003 (.001, .006)	.**024**	−.004 (−.006, −.001)	.**015**
BUN (U/L)	−.054 (−.097, −.011)	.**014**	−.054 (−.105, −.004)	.**034**	−.017 (−.064, .029)	.464	−.027 (−.076, .023)	.287
Phosphorus (mmol/L)	−.116 (−.511, .280)	.566	.036 (−.369, .440)	.863	−.688 (−1.006, −.371)	**<**.**001**	−.355 (−.649, −.060)	.**018**

Results are based on multivariable linear regression. Model 1 is unadjusted. Model 2 is adjusted for age, BMI, smoking, alcohol consumption, diet calcium, diabetes, hypertension, season, and calcium and vitamin D supplementations. Bold entries are a significant value of *P* < .05.

Abbreviations: ALP, alkaline phosphatase; BMI, body mass index; BUN, blood urea nitrogen.

### Relationships Between 24-Hour UCaE/Ucr and Bone Metabolism

As shown in Table 3, the 24-hour UCaE/Ucr was independently positively associated with 25(OH)D in both men (β = .014, *P* = .032) and women (β = .042, *P <* .001). Specifically, the 24-hour UCaE/Ucr was independently associated with PTH (β = −.006, *P =* .031), β-CTX (β = −.378, *P =* .021), and P1NP (β = −.005, *P =* .048) in men, but not in women.

**Table 3.** Association of PTH, 25(OH)D, and BTMs with ln-transformed 24-hour UCaE/Ucr by sex

Independent variables	Men				Women			
	Model 1		Model 2		Model 1		Model 2	
	β (95% CI)	*P* value	β (95% CI)	*P* value	β (95% CI)	*P* value	β (95% CI)	*P* value
PTH level								
Quartiles 1	Reference		Reference		Reference		Reference	
Quartiles 2	.179 (−.087, .444)	.187	.114 (−.151, .378)	.399	−.111 (−.344, .122)	.112	−.048 (−.257, .161)	.653
Quartiles 3	−.175 (−.441, .091)	.196	−.235 (−.499, .030)	.082	.055 (−.198, .308)	.382	.067 (−.161, .295)	.564
Quartiles 4	−.286 (−.552, −.021)	.035	−.310 (−.577, −.043)	.**023**	−.138 (−.358, .082)	.201	−.122 (−.320, .077)	.231
PTH (ng/mL)	−.006 (−.011, −.001)	.018	−.006 (−.011, −.001)	.**031**	−.007 (−.012, −.002)	.219	−.004 (−.008, .001)	.077
25(OH)D level								
Quartiles 1	Reference		Reference		Reference		Reference	
Quartiles 2	.338 (.076, .600)	.**012**	.233 (−.031, .496)	.084	.051 (−.160, .262)	.635	−.056 (−.257, .144)	.583
Quartiles 3	.279 (.21, .537)	.**034**	.165 (−.100, .431)	.222	.442 (.234, .650)	**<**.**001**	.256 (.052, .461)	.**014**
Quartiles 4	.551 (.280, .822)	**<**.**001**	.424 (.132, .717)	.**005**	1.252 (1.039, 1.465)	**<**.**001**	.850 (.625, 1.075)	**<**.**001**
25(OH)D (ng/mL)	.018 (.007, .030)	.**002**	.014 (.001, .026)	.**032**	.408 (.340, .476)	**<**.**001**	.042 (.031, .052)	**<**.**001**
β-CTX level								
Quartiles 1	Reference		Reference		Reference		Reference	
Quartiles 2	−.315 (−.579, −.051)	.**020**	−.306 (−.569, −.043)	.**023**	−.329 (−.559, .100)	.105	−.204 (−.413, .004)	.054
Quartiles 3	−.207 (−.475, .061)	.130	−.161 (−.433, .112)	.246	−.183 (−.414, .048)	.120	−.131 (−.379, .037)	.107
Quartiles 4	−.359 (−.629, −.089)	.**009**	−.277 (−.555, .001)	.050	−.053 (−.286, .179)	.652	−.124 (−.393, .028)	.089
β-CTX (ng/mL)	−.470 (−.812, −.129)	.**007**	−.378 (−.700, −.057)	.**021**	−.004 (−.077, .070)	.925	−.262 (−.640, .115)	.173
P1NP level								
Quartiles 1	Reference		Reference		Reference		Reference	
Quartiles 2	−.230 (−.496, .036)	.090	−.207 (−.477, .063)	.132	−.310 (−.543, −.078)	.**009**	−.188 (−.399, .023)	.081
Quartiles 3	−.269 (−.535, −.002)	.**048**	−.208 (−.484, −.069)	.141	−.258 (−.490, −.026)	.**030**	−.153 (−.364, .057)	.153
Quartiles 4	−.446 (−.713, −.179)	.**001**	−.427 (−.708, −.145)	.**003**	−.088 (−.320, .145)	.460	−.093 (−.303, .117)	.385
P1NP (ng/mL)	−.005 (−.009, −.001)	.**039**	−.005 (−.009, −.001)	.**048**	−.021 (−.095, .053)	.577	−.001 (−.004, .002)	.416

Results in this table are based on multivariable linear regression. Model 1 is unadjusted. Model 2 is adjusted for age, BMI, smoking, alcohol consumption, diet calcium, diabetes, hypertension, season, calcium and vitamin D supplementations. β-CTX and P1NP were divided into quartiles, with the first quartile having the lowest value and the fourth quartile having the highest value. Bold entries are a significant value of *P* < .05.

PTH level: (men: < 27.6, 27.6-35.6, 35.6-45.9, ≥ 45.9; women: < 27.8, 27.8-35.5, 35.5-42.3, ≥ 42.3). 25(OH)D level: (men: < 13, 13-18, 18-24, ≥ 24; women: <11, 11-15, 15-22, ≥ 22). β-CTX level: (men: <0.28, 0.28-0.42, 0.42-0.60, ≥ 0.60; women: < 0.24, 0.24-0.36, 0.36-0.53, ≥ 0.53). P1NP level: (men: < 32.4, 32.4-43.7, 43.7-59.0, ≥ 59.0; women: < 32.84, 32.84-42.62, 42.62-58.13, ≥ 58.13).

Abbreviations: 25(OH)D, 25-hydroxyvitamin D; β-CTX, type Ⅰ collagen-containing cross-linked C-telopeptide; P1NP, procollagen type Ⅰ N-propeptide; PTH, parathyroid hormone.

**Figure 2.**
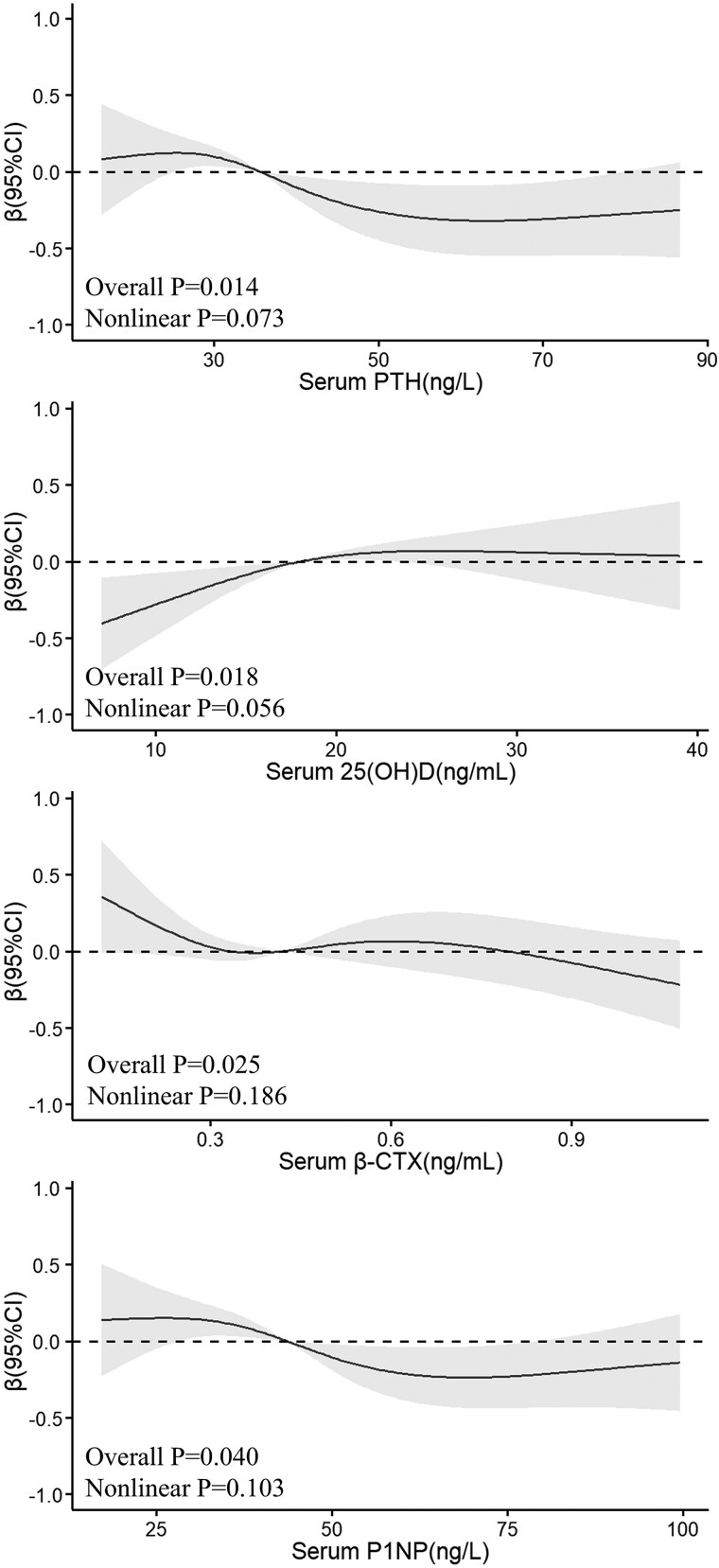
Relationship of 24-hour UCaE/Ucr with PTH, 25(OH)D, β-CTX, and P1NP in men. Restricted cubic splines were utilized to flexibly model the association between the 24-hour UCaE/Ucr and PTH, 25(OH)D, β-CTX, and P1NP adjusted for age, BMI, smoking, dietary calcium, season, calcium supplementation, and vitamin D supplementation. Abbreviations: 25(OH)D, 25-hydroxyvitamin D; β-CTX, type Ⅰ collagen-containing cross-linked C-telopeptide; P1NP, procollagen I type N-propeptide; PTH, parathyroid hormone; UCaE/Ucr, urinary calcium excretion/urinary creatinine.

**Figure 3.**
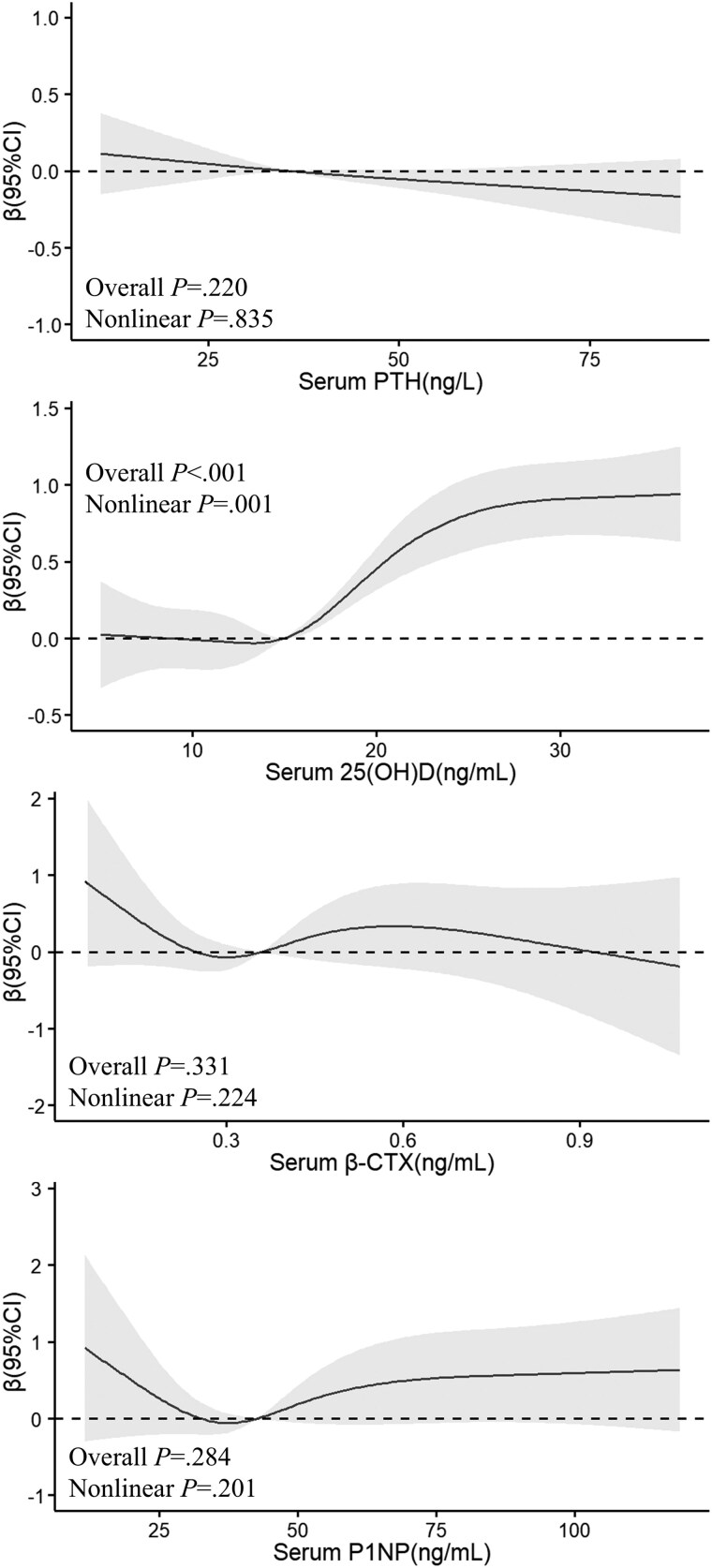
Relationship of 24-hour UCaE/Ucr with PTH, 25(OH)D, β-CTX and P1NP in women. Restricted cubic splines were utilized to flexibly model the association between the 24-hour UCaE/Ucr and PTH, 25(OH)D, β-CTX, and P1NP adjusted for age, BMI, smoking, dietary calcium, season, calcium supplementation, and vitamin D supplementation. Abbreviations: 25(OH)D, 25-hydroxyvitamin D; β-CTX, type Ⅰ collagen-containing cross-linked C-telopeptide; P1NP, procollagen I type N-propeptide; PTH, parathyroid hormone; UCaE/Ucr, urinary calcium excretion/urinary creatinine.

Figures 2 and 3 depict the restricted cubic splines for men and women, respectively. Men exhibit a significant negative nonlinear trend between PTH (*P* for overall = .014), β-CTX (*P* for overall = .025), and P1NP (*P* for overall = .040) with 24-hour UCaE/Ucr. A positive nonlinear relationship is noted for 25(OH)D (*P* for overall = .018) (Fig. 2). In women, as shown in Fig. 3, only 25(OH)D levels display a significant positive linear correlation with 24-hour UCaE/Ucr (*P* for overall < .001) (Fig. 3).

Subgroup analysis based on age, menopausal status, BMI, dietary calcium intake, and calcium supplement showed consistent results across all subgroups (Supplementary Table S2a and Supplementary Table S2b (22)). Specifically, we found that in the calcium supplement group and postmenopausal group, β-CTX levels were negatively correlated with 24-hour UCaE/Ucr.

### Prevalence of Hypercalciuria

**Figure 4.**
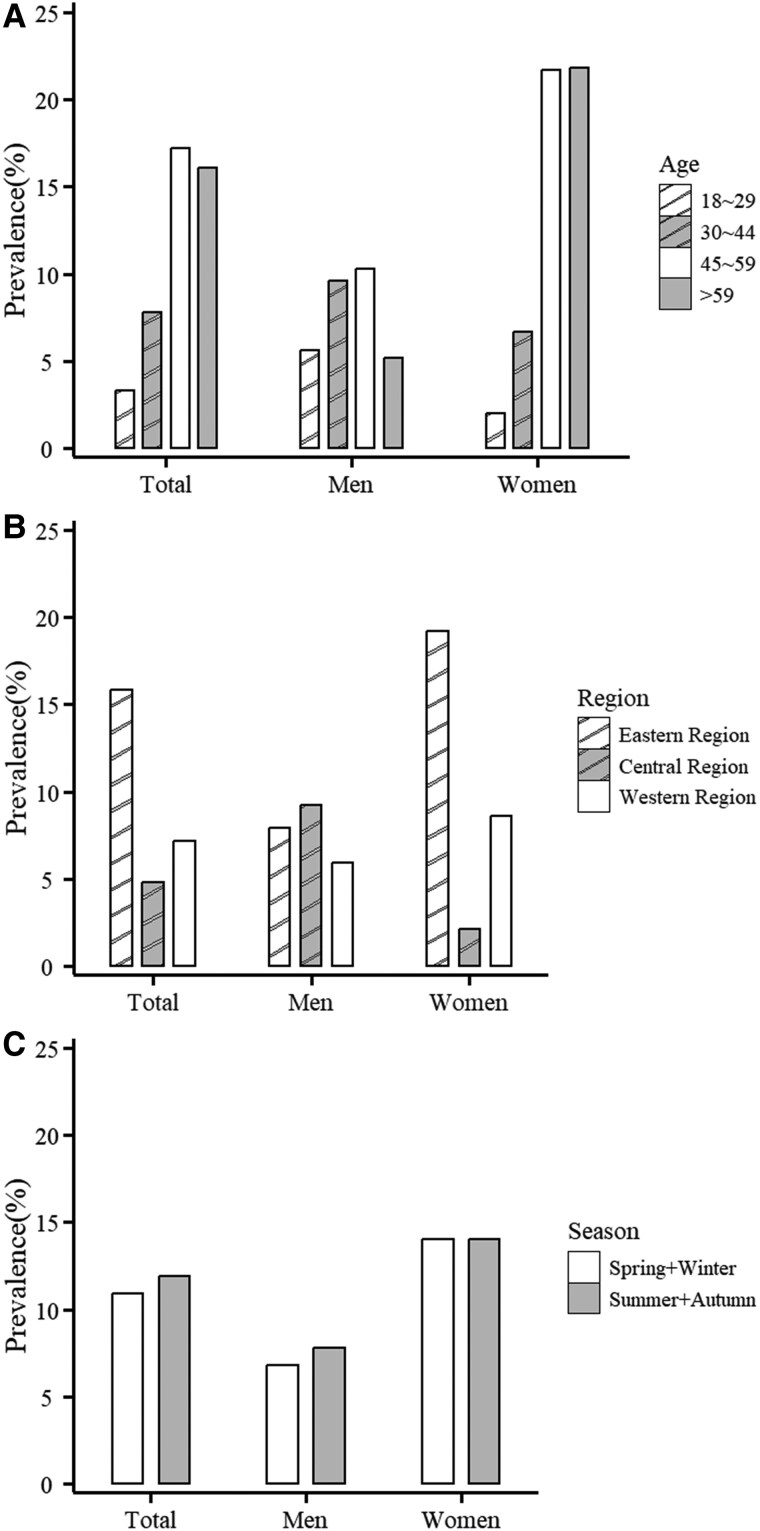
The rate of hypercalciuria by sex across (A) age strata, (B) regions, and (C) seasons.

To further understand the potential impact of hypercalciuria on overall health and its association with various risk factors, we conducted a detailed analysis of the prevalence of hypercalciuria and its association factors. The prevalence of hypercalciuria was 11.6% (144/1239) in the study population, with a marked disparity between genders: 7.5% (34/453) in men and 14.0% (110/786) in women. Statistically significantly differences in prevalence were noted across age groups and regions (*P* < .05 for all). Specifically, the prevalence rate was observed in 3.3% among those aged 18-29 years, 7.8% in those aged 30-44 years, 17.2% among those 45-59 years, and 16.1% in those aged ≥ 60 years (Fig. 4A). Regional disparities were also observed; the prevalence was 15.8% in Eastern China, 7.2% in Western China and 4.8% in Central China (*P* < .001) (Fig 4B). Seasonally, there was no statistically significant difference in the prevalence of hypercalcemia between different seasons (10.9% vs 11.9%, *P =* .633) (Fig. 4C).

## Discussion

This study provides a reference for the distribution of 24-hour UCaE in the Chinese population. The median 24-hour UCaE in the study population was 2.27 mmol, with men exhibiting a median of 2.24 mmol and women 2.28 mmol. Additionally, we observed a negative association between 24-hour UCaE and both β-CTX and P1NP in men, independent of age and other covariates; this association was not present in women. The prevalence of hypercalciuria was 11.6% in the overall population, with 7.5% in men and 14.0% in women.

Overall, the findings of our study indicated that the 24-hour UCaE reference intervals in our Chinese study are lower compared to those reported in European countries. For instance, a study from the Netherlands Medical Centre reported a 24-hour UCaE of 4.13 mmol in men and 3.52 mmol in women (23). However, when considering age strata in women, our study found similar 24-hour UCaE levels in elderly women, with a median of 2.46 mmol, compared with studies in the United States and among African American populations. A survey study involving 959 women in the United States reported a median of 24-hour UCaE of 3.29 mmol in elderly White women (6). Similarly, a cross-sectional study on postmenopausal women showed that the median 24-hour UCaE was 1.77 mmol in Black women, and 2.27 mmol in White women (24). Our findings were lower with the recommended reference range by clinical laboratories, where the normal range for 24-hour UCaE is 2.5 mmol to 7.5 mmol (25). Our results suggest that 6.7 mmol may be a better upper limit than 7.5 mmol for defining hypercalciuria in men, and in women, hypercalciuria would be defined as > 8.1 mmol.

An increasing number of researchers are emphasizing the need to focus on the factors influencing 24-hour UCaE and its relationship with bone health in the population. The regulation of 24-hour UCaE is influenced by 1,25-dihydroxy-vitamin D [1,25(OH)_2_D] and dietary factors (11, 26). The results of this study are similar to those of previous research. In elderly White women, 24-hour UCaE is significantly correlated with serum 25(OH)D levels and dietary calcium intake (24). These results are consistent with those of Rathod et al, who found that 24-hour UCaE was positively associated with 25(OH)D in women, independent of menopausal status and hormonal supplementation (27). Additionally, in elderly Black women, 24-hour UCaE is significantly but correlated with serum 1,25(OH)_2_D levels, dietary calcium intake, and calcium absorption (6, 28). Similarly, the InChianti study reported a positive correlation between UCaE and serum 25(OH)D levels in men (29). Our study corroborates these findings, showing that higher 24-hour UCaE is associated with higher serum 25(OH)D levels, even after adjusting for age, calcium supplements, vitamin D supplements, dietary calcium levels, and seasons.

This study explores the association between 24-hour UCaE and bone metabolism, we found the 24-hour UCaE/Ucr was independently associated with PTH, β-CTX, and P1NP in men, but not in women. The restrictive cubic splines curve showed negative nonlinear associations of PTH, β-CTX and P1NP with 24-hour UCaE/Ucr. Previous study showed lower serum PTH levels and higher blood calcium and 25(OH)D levels in individuals with higher 24-hour UCaE (5), suggesting increased 24-hour UCaE as a response to enhanced intestinal calcium absorption, possibly mediated by vitamin D. A possible explanatory mechanism for the observed relationship between 24-hour UCaE and bone metabolism is that a high 24-hour UCaE directly affects bone tissue, potentially leading to bone loss (30). Monocytes in patients with hypercalciuria often produce more interleukin-6, a pro-inflammatory cytokine that promotes the formation and activity of osteoclasts, leading to increased osteoclast activity and bone resorption (31, 32). As osteoclast activity increases, more bone calcium is released into the bloodstream, raising blood calcium levels (33). This study found a significant association between 24-hour UCaE and bone metabolism.

Bone turnover markers are enzymes and proteins secreted by osteoblasts or osteoclasts, primarily used to assess bone metabolic activity and bone health (34). These markers can reflect the activity of bone cells and provide information on bone tissue metabolism in a short period, predict fracture risk, and evaluate the early efficacy of anti-osteoporosis treatments, holding significant clinical importance (35). This study found a significant sex difference in the relationship between 24-hour UCaE and bone metabolism, with 24-hour UCaE being negatively associated with PTH, β-CTX, and P1NP in men, but not in women. This finding is consistent with previous research by Vezzoli et al, who conducted an epidemiological study involving 595 participants from the Tuscany region in Italy and reported a significant negative correlation between 24-hour UCaE and volumetric BMD in men, but no such relationship in women (12). One possible explanation for this observed sex difference is the differential regulation of renal calcium clearance by sex hormones. Higher 24-hour UCaE accelerates age-related bone loss in men, whereas the negative impact of calcium excretion on bones is minimal in women (36). This disparity is possibly due to estrogen, which plays a crucial role in maintaining bone quality and preventing osteoporosis (37, 38). Estrogen is a key hormonal regulator of bone metabolism, and estrogen deficiency is the most potent determinant of reduced BMD in postmenopausal women (39, 40). It inhibits osteoclast activity, thereby reducing calcium excretion and promoting calcium deposition in bones (41). Estrogen also regulates vitamin D metabolism, playing a crucial role in the absorption of calcium and phosphorus. Estrogen imbalances can disrupt calcium-phosphorus metabolism, heightening the risk of bone loss and soft tissue calcification (42). Additionally, animal studies have demonstrated that estrogen increases renal calcium reabsorption, while testosterone decreases it (29). Furthermore, a mechanistic study proposed that sex differences in UCaE might be due to varying activities of the enzymes CYP24A1 or CYP27B1 in men and women (36). Future studies are warranted to elucidate the mechanisms linking UCaE and bone metabolism and to better understand the sex-specific differences observed in these associations.

The overall prevalence of hypercalciuria was 11.6%, with 7.5% in men and 14.0% in women. These rates are different to those reported in previous studies in Western countries. Anita et al defined hypercalciuria as 0.1 mmol/kg per 24 hours and found a prevalence of 9.0% in men and 8.1% in women (38). While the prevalence in non-stone-forming men and women was 14-17% when using 24-hour UCaE > 7.49 mmol and > 6.24 mmol as the definitions of hypercalciuria in men and women, respectively (43). Furthermore, the prevalence of hypercalciuria varied across different age groups in our study. The highest prevalence was 10.3% in men aged 45 to 59 years and 21.8% in women aged ≥ 60 years.

However, the study has several limitations. First, participants were recruited continuously from 9 tertiary care hospitals, which may limit the generalizability of the study findings to the broader population. Second, only blood biochemistries and BTMs were assessed; BMD and thyroid-stimulating hormone were not measured. Third, the use of calcium and vitamin D supplements was based on self-reported questionnaires, and dietary habits were assessed based on participant recall rather than using prospective diaries, which may have introduced recall bias. Fourth, other confounding factors, such as physical activity and hydration status, were not measured. Fifth, although this study is a multicenter cross-sectional study, the sample size is still relatively small, which may lead to some insufficient statistical power on genetic factors associations. Sixth, the rather wide 90% CI around the upper limit is a limitation, related to the skewed distribution of the results. Despite these limitations, this study initially describes the distribution of 24-hour UCaE based on data from the Chinese population.

## Conclusion

In conclusion, this study established reference intervals for 24-hour UCaE in the Chinese population. Notably, 24-hour UCaE/Ucr was negatively correlated with PTH, β-CTX, and P1NP in men, while no such correlations were found in women, highlighting a significant sex difference. Future research studies are warranted to investigate the relationship between 24-hour UCaE and bone metabolism, focusing on elucidating the mechanisms underlying these sex-specific differences.

## Data Availability

Restrictions apply to the availability of some or all data generated or analyzed during this study to preserve patient confidentiality or because they were used under license. The corresponding author will on request detail the restrictions and any conditions under which access to some data may be provided.

## Clinical Trial Registration

This study was registered with CHICTR.ORG.CN (ChiCTR2200056577).

## Abbreviations

25(OH)D: 25-hydroxyvitamin D
ALP: alkaline phosphatase
ALT: alanine aminotransferase
β-CTX: type Ⅰ collagen-containing cross-linked C-telopeptide
BMD: bone mineral density
BMI: body mass index
BTM: bone turnover marker
BUN: blood urea nitrogen
P1NP: procollagen type 1 N-propeptide
PTH: parathyroid hormone
UCaE: urinary calcium excretion
Ucr: urinary creatinine
